# Supplementary material for: Trio-based whole exome sequencing in patients with suspected sporadic inborn errors of immunity: A retrospective cohort study
Source: eLife. 2022 Oct 17;11:e78469. doi: 10.7554/eLife.78469 (PMC9635875; doi:10.7554/eLife.78469)
Supplement: Figure 1—source code 1. [file elife-78469-fig1-code1.zip › Source-Code.html]

DNM\_code


# Filtering strategy and output generation for *de novo* variant analysis of trio-based whole exome sequencing datasets

## Retrieve data and create output folder

```
setwd("./DNM data")
TrioData <- list.files(path = ".", pattern = ".txt", full.names = TRUE)
dir.create("Output", recursive = TRUE)
```

## Filtering strategy applied to each trio-based WES dataset

```
for (a in TrioData[1:123]) {
  Trio <- read.delim(a, header = TRUE, sep = "\t")

  #Add column with ID of corresponding trio
    b <- str_split(a, "_hc")[[1]][[1]]
    c <- str_split(b, "/")[[1]][[2]]
    Trio$ID <- c(c)
    Trios <- Trio %>% relocate(ID)

  #Filter steps
    DeNovo <- Trios %>% filter(grepl("NOVO", De.novo.assessment))
    GenomicRegion <- DeNovo %>% filter(Gene.component %in% c("EXON_REGION", "SA_SITE_CANONICAL", "SD_SITE_CANONICAL", "CODING_SPLICE_SITE_REGION"))
    Rare <-GenomicRegion %>% filter(SNP.Frequency <= 0.1 & NonCausative…Frequency <= 0.1 & EXAC.AF <= 0.1 & AF_GoNL <= 0.5 & gnomAD.G.AF <= 0.1)
    QC <- Rare %>% filter(!grepl("LOW", De.novo.assessment)) %>% filter(variation.reads >= 10 & X..variation >= 20)
    NoSynonym <- QC %>% filter(Synonymous == "FALSE")

  setwd("Output")

  #All *de novo* variants (before filtering) in established IEI genes
    CheckIEI <- DeNovo %>% merge(GenePanel, by = "Gene.name")
    write.csv(CheckIEI, file = paste0(c, "-IEI.csv"), row.names = FALSE)

  #Candidate de novo variants
    write.csv(QC, file = paste0(c, "-DNM.csv"), row.names = FALSE)

  #Count candidate de novo variants
    Count_Candidates <- nrow(QC)
    d <- data.frame(Count_Candidates)
    d$ID <- c(c)
    e <- d %>% relocate(ID)
    write.csv(e, file = paste0(c, "-Count.csv"), row.names = FALSE)
    
  setwd("..") }

setwd("Output")
```

## Create one list of all *de novo* variants in known IEI genes of all 123 trio-based WES datasets

```
IndividualIEI <- list.files(path = ".", pattern = "-IEI", full.names = TRUE) %>%   lapply(read.csv, colClasses = c("character"))
AllIEI <- join_all(IndividualIEI, by = NULL, type = "full", match = "all")
write.xlsx(AllIEI, file = "IEI_DNM.xlsx")
```

## Create one list of candidate *de novo* variants of all 123 trio-based WES datasets

```
AllCandidates <- list.files(path = ".", pattern = "-DNM", full.names = TRUE) %>% lapply(read.csv, colClasses = c("character"))
AllC <- join_all(AllCandidates, by = NULL, type = "full", match = "all")
write.xlsx(AllC, file = "DNM.xlsx")
```

## Create one list of number of candidate *de novo* variants of all 123 trio-based WES datasets

```
IndividualCounts <- list.files(path = ".", pattern = "-Count", full.names = TRUE) %>% lapply(read.csv, colClasses = c("character"))
AllCounts <- join_all(IndividualCounts, by = NULL, type = "full", match = "all")
write.xlsx(AllCounts, file = "Counts.xlsx")
```
