# Supplementary material for: Trio-based whole exome sequencing in patients with suspected sporadic inborn errors of immunity: A retrospective cohort study
Source: eLife. 2022 Oct 17;11:e78469. doi: 10.7554/eLife.78469 (PMC9635875; doi:10.7554/eLife.78469)
Supplement: Figure 1—source data 1. [file elife-78469-fig1-data1.docx]

**Figure 1 – source data 1. List of 123 patient-parent trios with patient characteristics and whole exome sequencing performance statistics.**

| Patient nr. | Sex | Age range at sampling | Genetic ancestry | Phenotype (IUIS classification) | Median target coverage | % Targets ≥5x | % Targets ≥20x | Previously published |
| --- | --- | --- | --- | --- | --- | --- | --- | --- |
| 1 | M | 11-15 | Europe | SCID | 112 | 98.68 | 93.73 | Yes (28) |
| 2 | M | 16-20 | Europe | Bone marrow failure | 110 | 99.74 | 97.96 | Yes (10) |
| 3 | F | 31-35 | Europe | Immune dysregulation, syndromes with autoimmunity and others | 116 | 99.63 | 98.29 | No |
| 4 | M | 0-5 | Europe | Defect in intrinsic and innate immunity, bacterial and parasitic | 153 | 99.63 | 98.2 | Yes (10) |
| 5 | M | 0-5 | Europe | Predominantly antibody deficiency, hypogammaglobulinemia | 121 | 99.68 | 98.18 | No |
| 6 | M | 21-25 | Europe | Bone marrow failure | 134 | 99.2 | 95.08 | No |
| 7 | M | 46-50 | Europe | Autoinflammatory disorder | 100 | 99.66 | 97.88 | Yes (10) |
| 8 | M | 0-5 | Europe | CID, syndromal | 97 | 99.31 | 96.27 | Yes (10) |
| 9 | M | 6-10 | Europe | Predominantly antibody deficiency, hypogammaglobulinemia | 90 | 99.61 | 97.32 | Yes (10) |
| 10 | F | 0-5 | Europe | Immune dysregulation, HLH/EBV | 112 | 99.63 | 98.3 | Yes (10) |
| 11 | M | 0-5 | Europe | Immune dysregulation, HLH/EBV | 104 | 99.54 | 97.19 | Yes (10) |
| 12 | F | 11-15 | Middle East | CID, syndromal | 106 | 99.61 | 97.8 | Yes (10) |
| 13 | M | 16-20 | Europe | CID, syndromal | 139 | 99.54 | 97.56 | Yes (10) |
| 14 | M | 36-40 | Europe | Unclassified | 240 | 99.37 | 97.66 | Yes (10) |
| 15 | F | 0-5 | Europe | SCID | 86 | 98.8 | 93.7 | Yes (10) |
| 16 | F | 0-5 | Europe | Defect in intrinsic and innate immunity, MSMD and viral infection | 79 | 99.25 | 95.34 | Yes (10) |
| 17 | F | 6-10 | Europe | CID, syndromal | 109 | 99.53 | 97.59 | Yes (10) |
| 18 | F | 26-30 | Europe | CID, syndromal | 117 | 98.61 | 94.32 | Yes (10) |
| 19 | F | 6-10 | Europe | Autoinflammatory disorder | 119 | 99.2 | 96.11 | No |
| 20 | F | 26-30 | No Genetic Ancestry Info | Unclassified | 120 | 99.5 | 97.55 | No |
| 21 | M | 6-10 | Europe | Autoinflammatory disorder | 131 | 99.76 | 98.47 | Yes (10) |
| 22 | F | 31-35 | Europe | Immune dysregulation, autoimmunity and others | 118 | 99.4 | 97.06 | No |
| 23 | M | 11-15 | Middle East | Autoinflammatory disorder | 123 | 99.7 | 98.21 | Yes (10) |
| 24 | M | 0-5 | Europe | CID, syndromal | 119 | 99.4 | 95.92 | Yes (10) |
| 25 | F | 6-10 | Europe | Autoinflammatory disorder | 129 | 99.48 | 97.61 | Yes (10) |
| 26 | F | 0-5 | No Genetic Ancestry Info | Bone marrow failure | 174 | 99.16 | 95.82 | Yes (10) |
| 27 | M | 11-15 | Europe | Autoinflammatory disorder | 144 | 99.35 | 95.85 | No |
| 28 | F | 16-20 | Europe | Predominantly antibody deficiency, hypogammaglobulinemia | 110 | 99.48 | 97.15 | No |
| 29 | F | 26-30 | Europe | Autoinflammatory disorder | 110 | 99.3 | 96.44 | No |
| 30 | M | 6-10 | Europe | Defect in intrinsic and innate immunity, bacterial and parasitic | 102 | 99.27 | 95.47 | No |
| 31 | M | 6-10 | Africa | CID, syndromal | 108 | 99.51 | 96.61 | No |
| 32 | M | 11-15 | Europe | CID, syndromal | 127 | 99.55 | 97.28 | No |
| 33 | F | 36-40 | Europe | Immune dysregulation, autoimmunity and others | 115 | 99.35 | 96.79 | No |
| 34 | M | 0-5 | Europe | Defect in intrinsic and innate immunity, MSMD and viral infection | 119 | 99.15 | 94.49 | No |
| 35 | M | 11-15 | Europe | Defects in intrinsic and innate immunity | 114 | 99.2 | 94.45 | No |
| 36 | M | 0-5 | Europe | Bone marrow failure | 117 | 99.38 | 96.49 | No |
| 37 | M | 6-10 | Europe | Autoinflammatory disorder | 109 | 99.23 | 94.75 | No |
| 38 | F | 16-20 | Europe | Autoinflammatory disorder | 107 | 99.62 | 96.51 | No |
| 39 | F | 31-35 | Europe | Predominantly antibody deficiency, hypogammaglobulinemia | 134 | 99.33 | 96.83 | No |
| 40 | M | 11-15 | Europe | CID, syndromal | 121 | 99.42 | 96.54 | No |
| 41 | F | 51-55 | Europe | Bone marrow failure | 90 | 99.16 | 95.4 | No |
| 42 | M | 0-5 | Europe | Immune dysregulation, HLH/EBV | 106 | 99 | 94.14 | No |
| 43 | F | 0-5 | Europe | CID, syndromal | 106 | 98.5 | 92.92 | No |
| 44 | M | 16-20 | Europe | Predominantly antibody deficiency, hypogammaglobulinemia | 112 | 99.51 | 96.92 | No |
| 45 | M | 0-5 | No Genetic Ancestry Info | CID, syndromal | 225 | 99.04 | 95.09 | No |
| 46 | F | 16-20 | No Genetic Ancestry Info | Predominantly antibody deficiency, hypogammaglobulinemia | 180 | 99.18 | 95.25 | No |
| 47 | M | 16-20 | Europe | Predominantly antibody deficiency, hypogammaglobulinemia | 105 | 99.55 | 96.6 | No |
| 48 | M | 26-30 | No Genetic Ancestry Info | Predominantly antibody deficiency, hypogammaglobulinemia | 230 | 99.32 | 96.19 | No |
| 49 | M | 26-30 | Europe | Predominantly antibody deficiency, hypogammaglobulinemia | 125 | 99.7 | 97.52 | No |
| 50 | M | 6-10 | Europe | CID, syndromal | 102 | 99.54 | 96.62 | No |
| 51 | F | 0-5 | Europe | Autoinflammatory disorder | 125 | 99.58 | 97.78 | No |
| 52 | M | 11-15 | Europe | Predominantly antibody deficiency, hypogammaglobulinemia | 106 | 99.48 | 96.39 | No |
| 53 | F | 11-15 | Europe | Autoinflammatory disorder | 119 | 99.56 | 98.04 | No |
| 54 | F | 11-15 | Middle East | CID, syndromal | 117 | 99.51 | 97.48 | No |
| 55 | M | 6-10 | Europe | Predominantly antibody deficiency, hypogammaglobulinemia | 92 | 99.68 | 97.23 | No |
| 56 | F | 21-25 | Europe | Predominantly antibody deficiency, hypogammaglobulinemia | 109 | 99.56 | 97.52 | No |
| 57 | M | 0-5 | Europe | Autoinflammatory disorder | 103 | 99.67 | 97.25 | No |
| 58 | F | 21-25 | Europe | Unclassified | 113 | 99.39 | 96.88 | Yes (27) |
| 59 | M | 6-10 | Europe | Autoinflammatory disorder | 101 | 98.59 | 93.02 | No |
| 60 | M | 6-10 | Europe | Defects in intrinsic and innate immunity, MSMD and viral infection | 101 | 98.23 | 91.81 | No |
| 61 | M | 0-5 | No Genetic Ancestry Info | CID, syndromal | 21 | 86.15 | 48.53 | No |
| 62 | M | 0-5 | Europe | Immune dysregulation, autoimmunity and others | 125 | 98.93 | 95.02 | No |
| 63 | M | 6-10 | Europe | Immune dysregulation, autoimmunity and others | 105 | 98.63 | 93.33 | No |
| 64 | M | 0-5 | Europe | Immune dysregulation, autoimmunity and others | 120 | 98.9 | 94.92 | No |
| 65 | M | 0-5 | Europe | Defects in intrinsic and innate immunity | 105 | 98.53 | 93.02 | No |
| 66 | F | 0-5 | No Genetic Ancestry Info | CID, syndromal | 240 | 99.24 | 96.69 | No |
| 67 | M | 0-5 | Europe | Autoinflammatory disorder | 104 | 99.07 | 94.62 | No |
| 68 | F | 0-5 | Europe | Defects in intrinsic and innate immunity | 96 | 99.77 | 97.87 | No |
| 69 | M | 0-5 | Europe | CID, non-syndromal | 101 | 99.75 | 98.04 | No |
| 70 | F | 11-15 | Europe | Defects in intrinsic and innate immunity, MSMD and viral infection | 106 | 99.6 | 98.26 | No |
| 71 | F | 6-10 | Europe | Bone marrow failure | 105 | 99.63 | 98.34 | No |
| 72 | M | 0-5 | No Genetic Ancestry Info | Suspected SCID (low TRECs) | 148 | 99.42 | 96.14 | No |
| 73 | M | 0-5 | No Genetic Ancestry Info | Immune dysregulation, autoimmunity and others | 253 | 99.27 | 96.59 | No |
| 74 | M | 31-35 | Europe | Autoinflammatory disorder | 127 | 99.83 | 98.81 | No |
| 75 | F | 6-10 | Europe | Bone marrow failure | 99 | 99.09 | 98.92 | No |
| 76 | F | 0-5 | Europe | Immune dysregulation, autoimmunity and others | 90 | 99.6 | 97.64 | No |
| 77 | F | 0-5 | Europe | CID, syndromal | 116 | 99.66 | 98.32 | No |
| 78 | F | 6-10 | Europe | CID, syndromal | 111 | 99.64 | 98.1 | No |
| 79 | F | 6-10 | Europe | Congenital defect of phagocyte, neutropenia | 92 | 99.56 | 96.55 | No |
| 80 | F | 0-5 | No Genetic Ancestry Info | Suspected SCID (low TRECs) | 206 | 99.26 | 96.32 | No |
| 81 | F | 0-5 | Europe | Congenital defect of phagocyte, neutropenia | 110 | 99.66 | 98.07 | No |
| 82 | F | 26-30 | Europe | Predominantly antibody deficiency, hypogammaglobulinemia | 103 | 99.72 | 97.88 | No |
| 83 | F | 0-5 | No Genetic Ancestry Info | CID, non-syndromal | 181 | 98.91 | 95.1 | No |
| 84 | F | 0-5 | No Genetic Ancestry Info | Autoinflammatory disorder | 169 | 98.76 | 94.24 | No |
| 85 | F | 16-20 | Europe | Predominantly antibody deficiency, hypogammaglobulinemia | 90 | 99.46 | 96.32 | No |
| 86 | M | 6-10 | Middle East | Defect in intrinsic and innate immunity, MSMD and viral infection | 209 | 99.06 | 95.12 | No |
| 87 | M | 11-15 | Europe | Autoinflammatory disorder | 98 | 99.65 | 95.11 | No |
| 88 | F | 26-30 | Europe | Autoinflammatory disorder | 124 | 99.56 | 98.07 | No |
| 89 | F | 16-20 | Europe | CID, syndromal | 107 | 99.48 | 97.46 | No |
| 90 | M | 11-15 | Europe | Unclassified | 103 | 99.71 | 97.5 | No |
| 91 | F | 0-5 | Europe | Suspected SCID (low TRECs) | 116 | 97.67 | 90.26 | No |
| 92 | F | 0-5 | Europe | Suspected SCID (low TRECs) | 194 | 98.79 | 94.47 | No |
| 93 | F | 26-30 | Europe | Immune dysregulation, autoimmunity and others | 115 | 99.64 | 98.14 | No |
| 94 | F | 41-45 | Europe | Unclassified | 119 | 99.61 | 98 | No |
| 95 | M | 11-15 | Europe | Defects in intrinsic and innate immunity | 107 | 99.75 | 97.48 | No |
| 96 | M | 21-25 | Europe | CID, syndromal | 251 | 99.03 | 95.58 | No |
| 97 | F | 16-20 | Europe | Immune dysregulation, autoimmunity and others | 98 | 99.48 | 96.25 | No |
| 98 | M | 0-5 | Europe | SCID | 199 | 99.02 | 95.31 | No |
| 99 | F | 11-15 | Europe | Bone marrow failure | 179 | 98.55 | 93.77 | No |
| 100 | F | 21-25 | Europe | Autoinflammatory disorder | 111 | 99.46 | 97.78 | No |
| 101 | M | 0-5 | Middle East | Immune dysregulation, HLH/EBV | 128 | 98.73 | 92.71 | No |
| 102 | F | 11-15 | Middle East | Immune dysregulation, autoimmunity and others | 87 | 98.34 | 88.22 | No |
| 103 | F | 0-5 | Europe | Immune dysregulation, HLH/EBV | 121 | 99.51 | 97.3 | No |
| 104 | F | 0-5 | Europe | Defects in intrinsic and innate immunity | 247 | 98.9 | 95.47 | No |
| 105 | M | 31-35 | Europe | Defects in intrinsic and innate immunity, MSMD and viral infection | 111 | 99.27 | 98.79 | No |
| 106 | F | 11-15 | Europe | Immune dysregulation, autoimmunity and others | 111 | 99.53 | 97.71 | No |
| 107 | F | 0-5 | Europe | Defects in intrinsic and innate immunity | 98 | 99.4 | 97.09 | No |
| 108 | M | 21-25 | Europe | Bone marrow failure | 159 | 99.35 | 99.21 | No |
| 109 | M | 31-35 | Europe | Congenital defect of phagocyte, neutropenia | 115 | 99.71 | 97.99 | No |
| 110 | F | 0-5 | Europe | Immune dysregulation, autoimmunity and others | 92 | 99.41 | 96.82 | No |
| 111 | F | 0-5 | Europe | Defect in intrinsic and innate immunity, MSMD and viral infection | 101 | 99.52 | 97.5 | No |
| 112 | F | 6-10 | Europe | CID, syndromal | 101 | 99.51 | 97.52 | No |
| 113 | M | 0-5 | Europe | Autoinflammatory disorder | 96 | 99.62 | 96.94 | No |
| 114 | M | 6-10 | Europe | Immune dysregulation, autoimmunity and others | 114 | 99.33 | 99.16 | No |
| 115 | F | 0-5 | Europe | SCID | 151 | 99.1 | 98.96 | No |
| 116 | M | 0-5 | Central South Asia | Bone marrow failure | 125 | 99.34 | 99.17 | No |
| 117 | M | 0-5 | Europe | Congenital defect of phagocyte, neutropenia | 111 | 99.7 | 97.93 | No |
| 118 | F | 0-5 | Middle East | Immune dysregulation, autoimmunity and others | 86 | 99.11 | 98.82 | No |
| 119 | F | 11-15 | Europe | Autoinflammatory disorder | 120 | 99.57 | 97.98 | No |
| 120 | M | 11-15 | Europe | Congenital defect of phagocyte, functional defects | 167 | 99.37 | 99.23 | No |
| 121 | F | 0-5 | Middle East | Defects in intrinsic and innate immunity | 97 | 99.49 | 97.05 | No |
| 122 | M | 0-5 | Europe | Suspected SCID (low TRECs) | 114 | 99.36 | 99.16 | No |
| 123 | M | 0-5 | Europe | Autoinflammatory disorder | 98 | 99.61 | 96.45 | No |
|  |  |  |  | Average | 124.11 | 99.23 | 96.18 |  |

Abbreviations: IUIS = International Union of Immunological Societies; (S)CID = (severe) combined immunodeficiency; HLH = haemophagocytic lymphohistiocytosis; EBV = Epstein-Barr virus; MSMD = Mendelian susceptibility to mycobacterial disease; TREC = T cell receptor excision circle.
