# Supplementary material for: Trio-based whole exome sequencing in patients with suspected sporadic inborn errors of immunity: A retrospective cohort study
Source: eLife. 2022 Oct 17;11:e78469. doi: 10.7554/eLife.78469 (PMC9635875; doi:10.7554/eLife.78469)
Supplement: Figure 1—source data 2. [file elife-78469-fig1-data2.docx]

**Figure 1 – source data 2. List of all candidate rare, coding *de novo* variants found in this IEI cohort.**

| Patient nr. | Chromosome | Start position | Reference | Variant | % variation | Protein effect | Gene name |
| --- | --- | --- | --- | --- | --- | --- | --- |
| 1 | chr16 | 67968809 | C | T | 41.30 | missense variant | PSMB10 |
| 1 | chr19 | 30314571 | C | T | 46.67 | stop gained | CCNE1 |
| 1 | chr16 | 9858731 | G | A | 40.95 | synonymous variant | GRIN2A |
| 3 | chrX | 39933622 | G | A | 49.58 | missense variant | BCOR |
| 5 | chr4 | 140640926 | G | A | 48.41 | missense variant | MAML3 |
| 6 | chr4 | 3188351 | G | A | 21.19 | missense variant | HTT |
| 6 | chr7 | 107832197 | C | T | 26.53 | missense variant | NRCAM |
| 6 | chr8 | 10465340 | C | T | 21.57 | missense variant | RP1L1 |
| 6 | chr6 | 43039087 | T | C | 50.56 | synonymous variant | KLC4 |
| 6 | chr8 | 10465338 | T | A | 22.81 | synonymous variant | RP1L1 |
| 6 | chr8 | 10466094 | C | T | 25.14 | synonymous variant | RP1L1 |
| 8 | chr19 | 38028251 | G | A | 49.64 | missense variant | ZNF793 |
| 9 | chr11 | 8707228 | T | C | 52.27 | missense variant | RPL27A |
| 9 | chr16 | 613300 | G | A | 46.34 | synonymous variant | PRR35 |
| 10 | chr15 | 41865534 | G | A | 46.23 | missense variant | TYRO3 |
| 12 | chr9 | 33261159 | G | C | 46.67 | missense variant | BAG1 |
| 15 | chr12 | 133696890 | T | C | 50.00 | missense variant | ZNF891 |
| 16 | chr8 | 109226842 | C | T | 60.00 | missense variant | EIF3E |
| 16 | chr14 | 59757956 | A | C | 54.05 | missense variant | DAAM1 |
| 16 | chr14 | 65198823 | T | C | 45.45 | missense variant | PLEKHG3 |
| 16 | chr17 | 79892248 | G | A | 56.58 | missense variant | PYCR1 |
| 17 | chr17 | 48701721 | G | C | 44.62 | missense variant; splice region variant | CACNA1G |
| 18 | chr17 | 6023839 | G | A | 59.09 | missense variant | WSCD1 |
| 18 | chr20 | 31619534 | C | T | 41.71 | synonymous variant | BPIFB6 |
| 20 | chr10 | 64928256 | A | C | 44.07 | missense variant | JMJD1C |
| 21 | chr8 | 29994915 | C | T | 49.41 | missense variant | LEPROTL1 |
| 22 | chr1 | 109811902 | C | T | 64.10 | missense variant | CELSR2 |
| 22 | chr9 | 135521387 | G | A | 52.11 | synonymous variant | DDX31 |
| 23 | chr3 | 48502012 | G | C | 46.49 | missense variant | ATRIP |
| 25 | chrX | 48371004 | C | T | 50.68 | missense variant | PORCN |
| 25 | chr5 | 70806905 | CAA |  | 41.62 | stop gained; inframe deletion | BDP1 |
| 26 | chr14 | 33015396 | C | T | 41.84 | missense variant | AKAP6 |
| 26 | chr1 | 160160772 | T | C | 52.52 | synonymous variant | CASQ1 |
| 26 | chr1 | 170015893 | C | T | 47.27 | synonymous variant | KIFAP3 |
| 27 | chr16 | 29997683 | C | T | 52.08 | missense variant | TAOK2 |
| 27 | chr8 | 30700639 | G | A | 46.15 | synonymous variant | TEX15 |
| 28 | chr8 | 25292997 | C | T | 33.33 | missense variant | KCTD9 |
| 28 | chr8 | 144940811 | C | G | 34.88 | missense variant | EPPK1 |
| 29 | chr10 | 7318934 | TCT |  | 46.50 | inframe deletion | SFMBT2 |
| 29 | chr4 | 73186532 | C | A | 41.73 | missense variant | ADAMTS3 |
| 29 | chr2 | 46819659 | C | T | 52.11 | stop gained | PIGF |
| 29 | chr21 | 33732194 | G | A | 45.45 | stop gained | URB1 |
| 29 | chr1 | 12387862 | C | T | 47.06 | synonymous variant | VPS13D |
| 30 | chr19 | 33706718 | C | T | 53.13 | missense variant | SLC7A10 |
| 30 | chr5 | 35065629 | C | T | 49.52 | synonymous variant | PRLR |
| 31 | chr12 | 57920559 | CTCT |  | 54.14 | frameshift variant | MBD6 |
| 33 | chr5 | 64077751 | A | G | 45.45 | missense variant | CWC27 |
| 33 | chr10 | 26417401 | G | A | 51.72 | synonymous variant | MYO3A |
| 35 | chr14 | 92959959 | G | A | 33.73 | missense variant | SLC24A4 |
| 36 | chr6 | 170876017 | G | A | 49.62 | synonymous variant | TBP |
| 37 | chr6 | 43100171 | T | C | 42.95 | missense variant | PTK7 |
| 38 | chr1 | 161772035 | A | G | 53.03 | synonymous variant | ATF6 |
| 38 | chr15 | 42988057 | G | A | 59.49 | synonymous variant | STARD9 |
| 41 | chr1 | 16070935 | G | A | 48.67 | missense variant | TMEM82 |
| 41 | chr22 | 38211717 | C | T | 49.38 | missense variant | GCAT |
| 42 | chr2 | 175202175 | GGCGGCGGCAGCGGCGGCGGCGGCAGC |  | 55.56 | inframe deletion | SP9 |
| 42 | chr6 | 110944071 | C | T | 51.85 | synonymous variant | CDK19 |
| 43 | chr20 | 25596994 | A | G | 44.85 | missense variant | NANP |
| 46 | chr1 | 35824576 | A | C | 35.17 | missense variant | ZMYM4 |
| 46 | chr1 | 899890 | G | A | 46.05 | synonymous variant | KLHL17 |
| 49 | chr2 | 15769801 |  | T | 56.20 | frameshift variant | DDX1 |
| 49 | chr4 | 169845502 | GA |  | 49.53 | frameshift variant | PALLD |
| 49 | chr19 | 50000493 | AAG |  | 40.58 | inframe deletion | RPS11 |
| 49 | chr6 | 31749648 | G | A | 44.89 | missense variant | VARS1 |
| 49 | chr7 | 100279738 | C | T | 31.58 | missense variant | GIGYF1 |
| 50 | chr1 | 197129117 | C | A | 56.25 | missense variant | ZBTB41 |
| 50 | chr4 | 103866434 | C | A | 52.17 | missense variant | SLC9B1 |
| 50 | chrX | 54817426 | G | A | 97.03 | missense variant | ITIH6 |
| 50 | chr5 | 112769523 | C | T | 50.00 | synonymous variant | TSSK1B |
| 50 | chr20 | 23584162 | C | T | 53.62 | synonymous variant | CST9 |
| 51 | chr19 | 41386478 | G | T | 86.89 | synonymous variant | CYP2A7 |
| 52 | chr8 | 144874432 | C | T | 39.02 | missense variant | SCRIB |
| 52 | chr16 | 18830915 | T | C | 42.06 | missense variant | SMG1 |
| 53 | chr17 | 48070807 | T | C | 49.01 | missense variant | DLX3 |
| 53 | chr5 | 171295802 | T | C | 37.04 | splice acceptor variant | FBXW11 |
| 55 | chr11 | 129746725 | C | T | 49.48 | synonymous variant | NFRKB |
| 56 | chr15 | 76494597 | C | T | 47.30 | missense variant | TMEM266 |
| 57 | chr15 | 40587214 | A | T | 51.96 | missense variant | PLCB2 |
| 58 | chr16 | 67645905 | G | T | 43.70 | missense variant | CTCF |
| 59 | chr1 | 247587794 | C | T | 38.46 | missense variant | NLRP3 |
| 59 | chr9 | 19550157 | G | A | 54.82 | missense variant | SLC24A2 |
| 59 | chr17 | 78063997 | C | A | 29.58 | missense variant | CCDC40 |
| 59 | chr16 | 68056410 | C | T | 63.24 | synonymous variant | DDX28 |
| 60 | chr20 | 18794710 | G |  | 54.35 | frameshift variant | SCP2D1 |
| 60 | chr11 | 4870051 | G | A | 56.16 | missense variant | OR51S1 |
| 60 | chr10 | 99968165 | T | C | 44.58 | synonymous variant | R3HCC1L |
| 61 | chr17 | 77984488 | T | C | 43.75 | missense variant | TBC1D16 |
| 64 | chr16 | 83998933 | C | T | 53.25 | missense variant | OSGIN1 |
| 64 | chr3 | 49167806 | G | A | 48.82 | synonymous variant | LAMB2 |
| 65 | chr5 | 167645868 |  | C | 53.45 | frameshift variant | TENM2 |
| 65 | chr11 | 89135634 | TC |  | 64.29 | frameshift variant | NOX4 |
| 65 | chr1 | 44360053 | A | G | 45.24 | splice acceptor variant | ST3GAL3 |
| 66 | chr15 | 41102374 | C | T | 55.64 | missense variant | ZFYVE19 |
| 66 | chr17 | 72281342 | T | C | 55.90 | splice donor variant | DNAI2 |
| 66 | chr4 | 175898983 | A | G | 52.00 | synonymous variant | ADAM29 |
| 68 | chr17 | 42084846 | A | G | 50.60 | missense variant | NAGS |
| 68 | chr1 | 55168322 | T | C | 48.85 | synonymous variant | MROH7 |
| 70 | chr2 | 64779432 | C | A | 44.09 | missense variant | AFTPH |
| 72 | chr19 | 580774 | G |  | 56.07 | frameshift variant | BSG |
| 73 | chr10 | 70987078 |  | A | 47.33 | frameshift variant | HKDC1 |
| 73 | chr12 | 21471770 | G | C | 35.71 | missense variant | SLCO1A2 |
| 75 | chr1 | 78435621 | A | C | 46.67 | missense variant | FUBP1 |
| 76 | chr7 | 132571737 | G | C | 41.67 | missense variant | CHCHD3 |
| 76 | chr13 | 99047515 | G | A | 44.44 | missense variant | FARP1 |
| 76 | chr16 | 28837622 | G | A | 63.64 | missense variant | ATXN2L |
| 76 | chr19 | 38655406 | C | G | 40.43 | missense variant | SIPA1L3 |
| 77 | chrX | 117528078 | C | T | 45.59 | missense variant | WDR44 |
| 78 | chr7 | 151860074 | A |  | 46.79 | frameshift variant | KMT2C |
| 78 | chr1 | 43913308 | C | A | 42.45 | synonymous variant | SZT2 |
| 78 | chr8 | 135545120 | G | A | 49.15 | synonymous variant | ZFAT |
| 80 | chr15 | 90347487 | G | C | 56.15 | missense variant | ANPEP |
| 82 | chr2 | 215843642 | A | G | 59.63 | synonymous variant | ABCA12 |
| 83 | chr7 | 100282517 | C |  | 47.95 | frameshift variant; splice region variant | GIGYF1 |
| 83 | chr13 | 103382492 | A | G | 43.30 | missense variant | CCDC168 |
| 83 | chr15 | 63966552 | T | C | 49.02 | missense variant | HERC1 |
| 83 | chr16 | 89265147 | G | A | 54.52 | stop gained | SLC22A31 |
| 84 | chr3 | 101117774 | G | A | 47.62 | missense variant | SENP7 |
| 85 | chr2 | 179497010 | G | A | 51.72 | synonymous variant | TTN |
| 85 | chr13 | 76407282 | C | T | 49.62 | synonymous variant | LMO7 |
| 86 | chr16 | 75646363 | G | A | 50.85 | missense variant | ADAT1 |
| 86 | chr13 | 111142071 | C | T | 35.64 | synonymous variant | COL4A2 |
| 90 | chr19 | 40889864 | G | A | 39.39 | synonymous variant | HIPK4 |
| 91 | chr1 | 153789892 | G | A | 48.31 | missense variant | GATAD2B |
| 91 | chr22 | 43230306 | G | A | 36.36 | missense variant | ARFGAP3 |
| 92 | chr2 | 21363959 | G | A | 44.44 | missense variant | TDRD15 |
| 92 | chr21 | 19653534 | T | C | 40.76 | missense variant | TMPRSS15 |
| 95 | chr8 | 42179639 | G | A | 33.96 | synonymous variant | IKBKB |
| 96 | chr9 | 34256995 | T | A | 45.58 | synonymous variant | KIF24 |
| 97 | chr19 | 43376039 | C | T | 44.87 | missense variant | PSG1 |
| 98 | chr19 | 40719909 |  | G | 21.03 | frameshift variant | MAP3K10 |
| 98 | chr2 | 238249487 | T | G | 47.13 | missense variant | COL6A3 |
| 98 | chr6 | 43039601 | T | C | 55.00 | synonymous variant | KLC4 |
| 100 | chr2 | 186669876 | C | A | 39.10 | synonymous variant | FSIP2 |
| 101 | chr2 | 238004500 | A | G | 61.33 | missense variant | COPS8 |
| 101 | chr17 | 78341841 | T | C | 53.04 | missense variant | RNF213 |
| 102 | chr1 | 19609317 | C | T | 41.38 | missense variant | AKR7A3 |
| 102 | chr1 | 19609318 | C | T | 40.87 | synonymous variant | AKR7A3 |
| 104 | chr2 | 71163167 | C | T | 42.12 | missense variant | ATP6V1B1 |
| 105 | chr17 | 39240792 |  | CTAGCTGCTGCATCT | 23.29 | inframe insertion | KRTAP4-7 |
| 106 | chr1 | 155629606 | G | A | 40.72 | missense variant | YY1AP1 |
| 106 | chr18 | 47777275 | T | G | 23.96 | missense variant | CFAP53 |
| 107 | chr17 | 55957032 | G | A | 52.13 | missense variant | CUEDC1 |
| 108 | chr4 | 1959681 | G | T | 40.50 | missense variant | NSD2 |
| 108 | chr4 | 1959687 | G | T | 40.48 | missense variant | NSD2 |
| 108 | chr9 | 38414121 | A | G | 44.90 | synonymous variant | IGFBPL1 |
| 109 | chr20 | 5081567 | C | T | 51.22 | missense variant | TMEM230 |
| 110 | chr10 | 75528852 | TGAC |  | 58.33 | frameshift variant | SEC24C |
| 110 | chr14 | 93119129 | C | T | 48.18 | missense variant | RIN3 |
| 111 | chr10 | 70644299 | C | A | 42.61 | missense variant | STOX1 |
| 111 | chr17 | 4440216 | G | A | 51.52 | synonymous variant | SPNS2 |
| 112 | chr4 | 144468021 | A | G | 52.63 | synonymous variant | SMARCA5 |
| 113 | chr14 | 63863401 | C | G | 45.31 | missense variant | PPP2R5E |
| 113 | chr2 | 73680020 | A | G | 52.98 | synonymous variant | ALMS1 |
| 114 | chr17 | 39240792 |  | CTAGCTGCTGCATCT | 58.33 | inframe insertion | KRTAP4-7 |
| 115 | chr3 | 89521721 | C | T | 25.57 | missense variant | EPHA3 |
| 115 | chr18 | 7026058 | G | A | 49.12 | synonymous variant | LAMA1 |
| 116 | chr8 | 144999832 | A | C | 48.44 | missense variant | PLEC |
| 118 | chr1 | 25256227 | C | T | 47.57 | missense variant | RUNX3 |
| 118 | chr4 | 151770608 | A | C | 21.74 | missense variant | LRBA |
| 118 | chr19 | 40711861 | A | G | 26.79 | missense variant | MAP3K10 |
| 118 | chr9 | 140139542 | T | G | 33.33 | splice region variant; synonymous variant | FAM166A |
| 118 | chr4 | 88537012 | C | T | 20.35 | synonymous variant | DSPP |
| 119 | chr18 | 72997837 | ACA |  | 21.05 | inframe deletion | TSHZ1 |
| 119 | chr15 | 75641602 | G | A | 59.46 | missense variant | NEIL1 |
| 119 | chr11 | 65423234 | C | T | 54.62 | splice acceptor variant | RELA |
| 120 | chr1 | 32280068 | G | A | 50.00 | synonymous variant | SPOCD1 |
| 120 | chr19 | 15535770 | T | A | 53.85 | synonymous variant | WIZ |
| 121 | chr5 | 37364086 | T | C | 47.06 | missense variant; splice region variant | NUP155 |
| 122 | chr1 | 53387237 | C | T | 48.89 | missense variant | ECHDC2 |
| 123 | chr1 | 888593 | G | C | 51.88 | missense variant | NOC2L |
| 123 | chr2 | 27604480 | C | G | 43.24 | missense variant | PPM1G |
| 123 | chr17 | 60743473 | G | T | 55.77 | missense variant | MRC2 |
