# Supplementary material for: Trio-based whole exome sequencing in patients with suspected sporadic inborn errors of immunity: A retrospective cohort study
Source: eLife. 2022 Oct 17;11:e78469. doi: 10.7554/eLife.78469 (PMC9635875; doi:10.7554/eLife.78469)
Supplement: Figure 1—source data 3. — The total amount of candidate de novo variants was retrieved from our cohort, consisting of 123 individuals. All identified de novo variants with at least 20% variation reads of the Kaplanis et al., 2020. cohort were obtained. The amount of predicted loss-of-function, synonymous and non-synonymous de novo single nucleotide variants or small insertion-deletions were extracted from both cohorts. The total amount of all, predicted loss-of-function, synonymous and non-synonymous de novo variants were divided by the respective cohort size to obtain the average number of respective variants per individual for each cohort. [file elife-78469-fig1-data3.docx]

**Figure 1 – source data 3. *De novo* variant rate and distribution of *de novo* variant types across our IEI cohort in comparison to a reference cohort from Kaplanis *et al.* (8).** The total amount of candidate *de novo* variants was retrieved from our cohort, consisting of 123 individuals. All identified *de novo* variants with at least 20% variation reads of the Kaplanis *et al.* cohort were obtained (8). The amount of predicted loss-of-function, synonymous and non-synonymous *de novo* single nucleotide variants or small insertion-deletions were extracted from both cohorts. The total amount of all, predicted loss-of-function, synonymous and non-synonymous *de novo* variants were divided by the respective cohort size to obtain the average number of respective variants per individual for each cohort.

|  | IEI cohort | Kaplanis *et al.* cohort (8) |
| --- | --- | --- |
| Number of individuals | 123 | 31,058 |
| All *de novo* SNVs or small indels |  |  |
| Total | 172 | 44,742 |
| Per individual | 1.40 | 1.44 |
| *De novo* predicted loss-of-function SNVs or small indels |  |  |
| Total | 23 | 7,196 |
| Per individual | 0.19 | 0.23 |
| *De novo* synonymous SNVs or small indels |  |  |
| Total | 48 | 8,868 |
| Per individual | 0.39 | 0.29 |
| *De novo* non-synonymous SNVs or small indels |  |  |
| Total | 101 | 28,678 |
| Per individual | 0.82 | 0.92 |

Abbreviations: IEI = inborn errors of immunity; SNV = single nucleotide variant; indel = insertion-deletion.
