# Supplementary material for: Trio-based whole exome sequencing in patients with suspected sporadic inborn errors of immunity: A retrospective cohort study
Source: eLife. 2022 Oct 17;11:e78469. doi: 10.7554/eLife.78469 (PMC9635875; doi:10.7554/eLife.78469)
Supplement: Table 1—source data 1. — The table displays information on inherited single nucleotide variants and small insertion-deletions or copy number variants that were identified after diagnostic in silico gene panel and/or exome-wide analysis prior to the systematic DNV analysis in this study. [file elife-78469-table1-data1.zip › Table 1 - source data 1.docx]

**Table 1 – source data 1. List of patient-parent trios with variants identified in genes outside the diagnostic IEI gene panel, or classified as risk factors, carriership or variants of uncertain significance.** The table displays information on inherited single nucleotide variants and small insertion-deletions or copy number variants that were identified after diagnostic *in silico* gene panel and/or exome-wide analysis prior to the systematic DNV analysis in this study.

| Patient nr. | Sex | Age range at sampling | Phenotype (IUIS classification) | Variant | | ACMG classification | Clinical significance | | Comments | | |
| --- | --- | --- | --- | --- | --- | --- | --- | --- | --- | --- | --- |
| 1 | M | 11-15 | SCID | FLG Chr1(GRCh37):g.152285861G>A  NM_002016.1:c.1501C>T p.(Arg501*) | | Pathogenic | Ichthyosis vulgaris  (OMIM #146700) | | Additional *de novo* SNV  (Table 2B) | | |
|  |  |  |  | arr[GRCh37] 16q21qter(?_qter)x2 | | - | Homozygous mosaicism | |  |  |  |
| 3 | F | 31-35 | Immune dysregulation, syndromes with autoimmunity and others | EYS Chr6(GRCh37):g.65300805G>C  NM_001142800.1:c.4955C>G p.(Ser1652*) | | Pathogenic | Retinitis pigmentosa 25  (OMIM #602772) | |  | | |
|  |  |  |  | EYS Chr6(GRCh37):g.64430943A>T  NM_001142800.1:c.8984T>A p.(Ile2995Asn) | | Likely pathogenic |  |  |  |  |  |
|  |  |  |  | FGB Chr4(GRCh37):g.155486984C>T  NM_005141.4:c.139C>T p.(Arg47*) | | Pathogenic | Carriership  (htz, OMIM #202400) | |  |  |  |
| 6 | M | 21-25 | Bone marrow failure | PTCH1 Chr9(GRCh37):g.98231067_98231068delinsAA NM_000264.3:c.2215_2216delinsTT p.(His739Phe) | | Uncertain significance | Uncertain significance  (htz, OMIM #109400) | |  | | |
| 7 | M | 46-50 | Autoinflammatory disorder | C7 Chr5(GRCh37):g.40959614_40959616del  NM_000587.2:c.1553_1555del p.(Thr518del) | | Uncertain significance | Carriership  (htz, OMIM #610102) | |  | | |
| 16 | F | 0-5 | Defect in intrinsic and innate immunity, MSMD and viral infection | IRAK4 Chr12(GRCh37):g.44165145C>T  NM_001114182.2:c.284C>T p.(Ala95Val) | | Uncertain significance | Carriership  (htz, OMIM #607676) | |  | | |
| 21 | M | 6-10 | Autoinflammatory disorder | DNASE1 Chr16(GRCh37):g.3707343G>C  NM_005223.3:c.704+1G>C p.? | | Likely pathogenic | Risk factor  (htz, OMIM #152700) | |  | | |
|  |  |  |  | MUC2 Chr11(GRCh37):g.1078647G>C  ENST00000441003.2:c.855G>C p.(Trp285Cys) | | Uncertain significance | Uncertain significance (htz) | |  | | |
|  |  |  |  | seq[GRCh37] del(17)(q25.3qter)  NC_000017.10:g.(80544076_80544938)_qterdel | | Pathogenic | Uncertain significance (htz) | | *De novo* CNV Patients with overlapping (larger) CNVs have been described without a congruent phenotype (46) | | |
| 23 | M | 11-15 | Autoinflammatory disorder | TERT Chr5(GRCh37):g.1278865G>A  NM_198253.2:c.2177C>T p.(Thr726Met) | | Uncertain significance | Uncertain significance  (htz, OMIM #613989) | |  | | |
|  |  |  |  | NCF2 Chr1(GRCh37):g.183538298G>A  NM_000433.3:c.692C>T p.(Pro231Leu) | | Uncertain significance | Carriership  (htz, OMIM #233710) | |  | | |
| 32 | M | 11-15 | CID, syndromal | DOCK8 Chr9(GRCh37):g.304661C>T  NM_203447.3:c.485C>T p.(Thr162Met) | | Uncertain significance | Carriership  (htz, OMIM #243700) | |  | | |
| 40 | M | 11-15 | CID, syndromal | MYBPC3 Chr11(GRCh37):g.47353661del  NM_000256.3:c.3776del p.(Gln1259fs) | | Pathogenic | Hypertrophic cardiomyopathy 4  (OMIM #115197) | |  | | |
| 44 | M | 16-20 | Predominantly antibody deficiency, hypogammaglobulinemia | TNFRSF13B Chr17(GRCh37):g.16843729G>T  NM_012452.2:c.542C>A p.(Ala181Glu) | | Likely pathogenic | Risk factor  (htz, OMIM #240500) | |  | | |
|  |  |  |  | JAGN1 Chr3(GRCh37):g.9932407A>G  NM_032492.3:c.1A>G p.? | | Uncertain significance | Carriership  (htz, OMIM #616022) | |  | | |
| 45 | M | 0-5 | CID, syndromal | PARN Chr16(GRCh37):g.14721009G>A  NM_002582.3:c.281C>T p.(Pro94Leu) | | Uncertain significance | Uncertain significance  (hmz, OMIM #616353) | |  | | |
| 54 | F | 11-15 | CID, syndromal | ADAM17 Chr2(GRCh37):g.9666240T>A  NM_003183.5:c.753A>T p.(Leu251Phe) | | Uncertain significance | Uncertain significance  (hmz, OMIM #614328) | |  | | |
| 55 | M | 6-10 | Predominantly antibody deficiency, hypogammaglobulinemia | NOD2 Chr16(GRCh37):g.50746164C>T  NM_022162.2:c.2342C>T p.(Ala781Val) | | Uncertain significance | Risk factor  (htz, OMIM #266600) | |  | | |
| 56 | F | 21-25 | Predominantly antibody deficiency, hypogammaglobulinemia | TNFRSF13B Chr17(GRCh37):g.16843729G>T  NM_012452.2:c.542C>A p.(Ala181Glu) | | Likely pathogenic | Risk factor  (htz, OMIM #240500) | |  | | |
|  |  |  |  | TNFRSF13B Chr17(GRCh37):g.16852187A>G  NM_012452.2:c.310T>C p.(Cys104Arg) | | Likely pathogenic | Risk factor  (htz, OMIM #240500) | |  | | |
| 68 | F | 0-5 | Defects in intrinsic and innate immunity | TNFRSF13B Chr17(GRCh37):g.16852187A>G  NM_012452.2:c.310T>C p.(Cys104Arg) | | Likely pathogenic | Risk factor  (htz, OMIM #240500) | |  | | |
| 69 | M | 0-5 | CID, non-syndromal | seq[GRCh37] dup(22)(q11.21q11.21)  NC_000022.10:g.(18775421_18893960)_(21414845_21576183)dup | | Pathogenic | Chromosome 22q11.2 microduplication syndrome (OMIM #608363) | | *De novo* CNV | | |
| 76 | F | 0-5 | Immune dysregulation, autoimmunity and others | ATM Chr11(GRCh37):g.108123641T>G  NM_000051.3:c.1898+2T>G p.? | | Pathogenic | Carriership  (htz, OMIM #208900) | |  | | |
| 80 | F | 0-5 | Suspected SCID (low TRECs) | JAK3 Chr19(GRCh37):g.17953972C>T  NM_000215.3:c.430G>A p.(Asp144Asn) | | Uncertain significance | Uncertain significance  (ch, OMIM #600802) | |  | | |
|  |  |  |  | JAK3 Chr19(GRCh37):g.17955108C>T  NM_000215.3:c.119G>A p.(Arg40His) | |  |  |  |  |  |  |
| 85 | F | 16-20 | Predominantly antibody deficiency, hypogammaglobulinemia | TNXB Chr6(GRCh37):g.32049159_32049162dup  NM_001365276.1:c.4025_4028dup p.(Val1344fs) | | Pathogenic | Classic-like Ehlers-Danlos syndrome  (OMIM #606408) | |  | | |
|  |  |  |  | seq[GRCh37] dup(17)(p12p12) NC_000017.10:g.(14080637_14095281)_(15457168_15458539)dup | | Pathogenic | Charcot-Marie-Tooth disease type 1A  (OMIM #118220) | |  |  |  |
| 100 | F | 21-25 | Autoinflammatory disorder | GABRG2 Chr5(GRCh37):g.161580318C>T  NM_198903.2:c.1492C>T p.(Pro498Ser) | | Uncertain significance | Uncertain significance  (htz, OMIM #607681) | |  | | |
| 101 | M | 0-5 | Immune dysregulation, HLH/EBV | TACO1 Chr17(GRCh37):g.61678707C>T  NM_016360.3:c.265C>T p.(Arg89Cys) | Uncertain significance | | | Uncertain significance  (htz, OMIM #619052) | |  |  |
|  |  |  |  | TACO1 Chr17(GRCh37):g.61683701A>C  NM_016360.3:c.416A>C p.(Glu139Ala) |  |  |  |  |  |  |  |
| 103 | F | 0-5 | Immune dysregulation, HLH/EBV | PKLR Chr1(GRCh37):g.155261636C>T  NM_000298.5:c.1529G>A p.(Arg510Gln) | Pathogenic | | | Pyruvate kinase deficiency  (OMIM #266200) | |  |  |
|  |  |  |  | PKLR Chr1(GRCh37):g.155265334A>T  NM_000298.5:c.401T>A p.(Val134Asp) | Pathogenic | | |  |  |  |  |
| 112 | F | 6-10 | CID, syndromal | TNFRSF13B Chr17(GRCh37):g.16852187A>G  NM_012452.2:c.310T>C p.Cys104Arg | Likely pathogenic | | | Risk factor  (htz, OMIM #240500) | |  |  |
| 115 | F | 0-5 | SCID | seq[GRCh37] dup(X)(q13.1q13.1) NC_000023.10:g.(70391543_70443557)_(70838299_70887653)dup | Uncertain significance | | | Uncertain significance (htz) | | *De novo* CNV |  |

Abbreviations: IUIS = International Union of Immunological Societies; ACMG = American College of Medical Genetics and Genomics; (S)CID = (severe) combined immunodeficiency; MSMD = mendelian susceptibility to mycobacterial disease; TREC = T cell receptor excision circle; HLH = haemophagocytic lymphohistiocytosis; EBV = Epstein-Barr virus; OMIM = Online Mendelian Inheritance in Man; htz = heterozygous; hmz = homozygous; ch = compound heterozygous; SNV = single nucleotide variant; CNV = copy number variant.
