# Supplementary figures and images for: Trio-based whole exome sequencing in patients with suspected sporadic inborn errors of immunity: A retrospective cohort study
Source: eLife. 2022 Oct 17;11:e78469. doi: 10.7554/eLife.78469 (PMC9635875; doi:10.7554/eLife.78469)

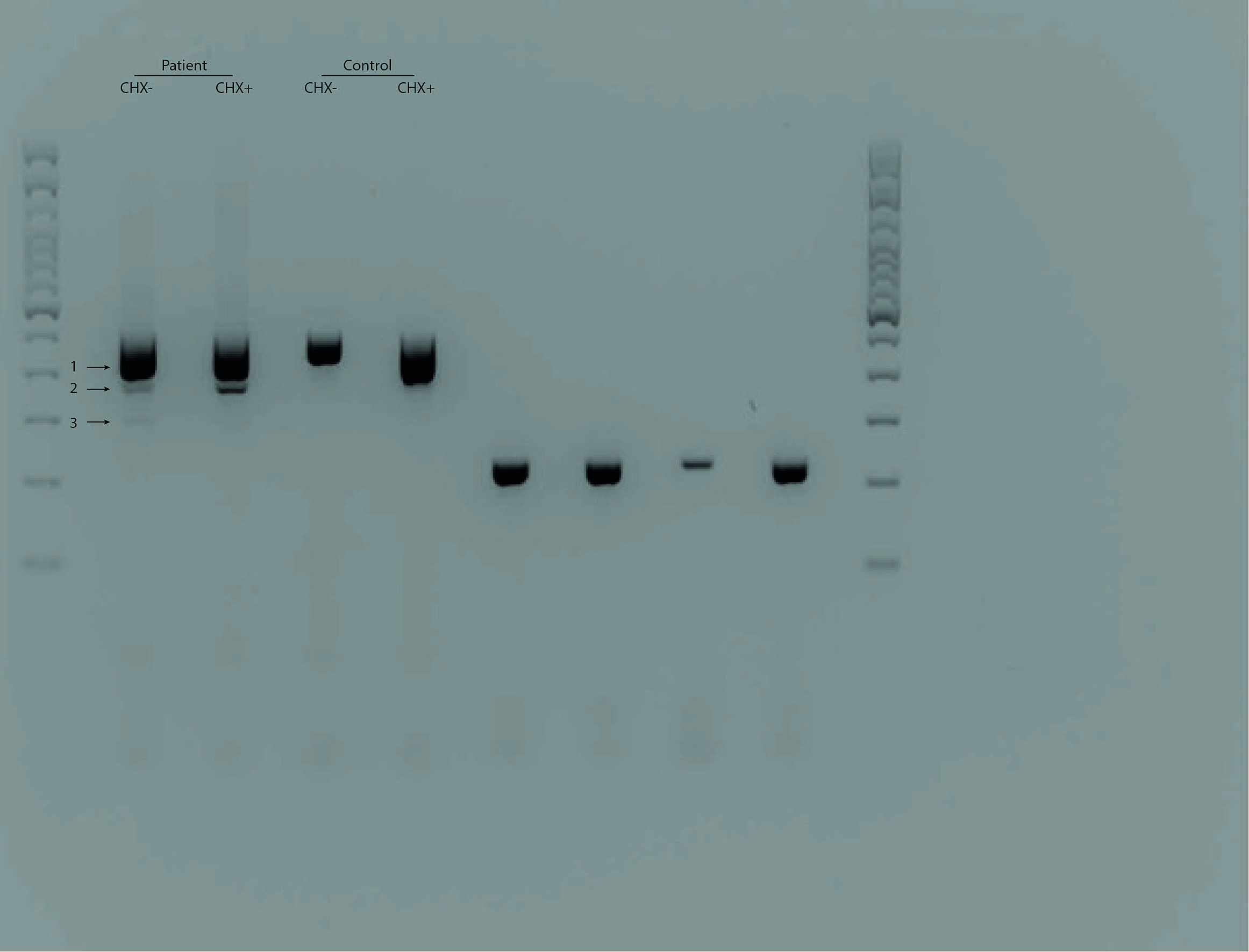

Supplement: Figure 2—source data 2. [file elife-78469-fig2-data2.zip › Figure 2 - source data 2 - raw gel labels.jpg]

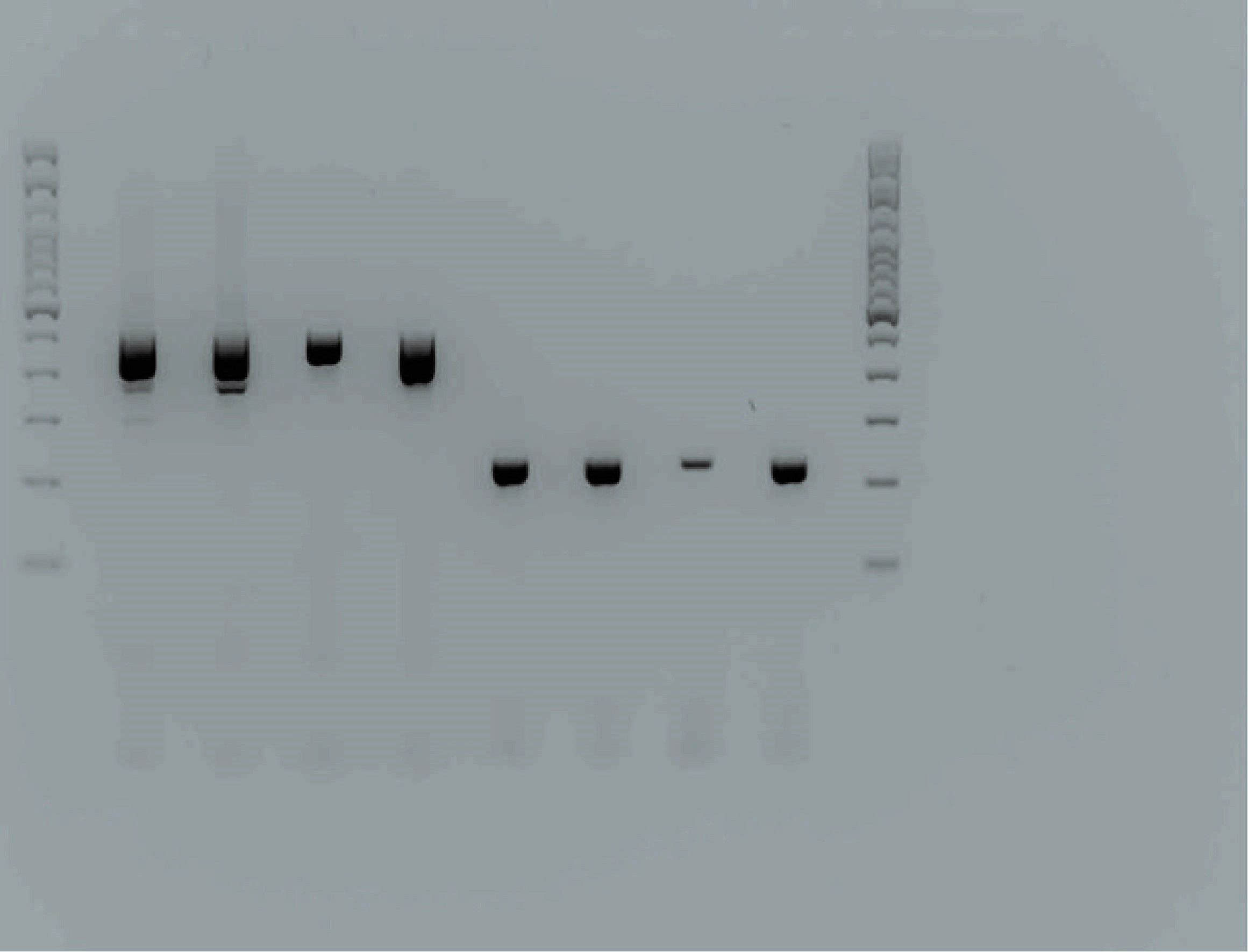

Supplement: Figure 2—source data 2. [file elife-78469-fig2-data2.zip › Figure 2 - source data 2 - raw gel.jpg]
